# Pre-Transplant CDKN2A Expression in Kidney Biopsies Predicts Renal Function and Is a Future Component of Donor Scoring Criteria

**DOI:** 10.1371/journal.pone.0068133

**Published:** 2013-07-04

**Authors:** Marc Gingell-Littlejohn, Dagmara McGuinness, Liane M. McGlynn, David Kingsmore, Karen S. Stevenson, Christian Koppelstaetter, Marc J. Clancy, Paul G. Shiels

**Affiliations:** 1 University of Glasgow, College of Medical, Veterinary and Life Sciences, Institute of Cancer Sciences, Glasgow, United Kingdom; 2 Transplant Unit, Western Infirmary, Glasgow, United Kingdom; 3 Division of Nephrology, Department of Internal Medicine, Medical University Innsbruck, Innsbruck, Austria; INSERM, France

## Abstract

CDKN2A is a proven and validated biomarker of ageing which acts as an off switch for cell proliferation. We have demonstrated previously that CDKN2A is the most robust and the strongest pre-transplant predictor of post- transplant serum creatinine when compared to “Gold Standard” clinical factors, such as cold ischaemic time and donor chronological age. This report shows that CDKN2A is better than telomere length, the most celebrated biomarker of ageing, as a predictor of post-transplant renal function. It also shows that CDKN2A is as strong a determinant of post-transplant organ function when compared to extended criteria (ECD) kidneys. A multivariate analysis model was able to predict up to 27.1% of eGFR at one year post-transplant (p = 0.008). Significantly, CDKN2A was also able to strongly predict delayed graft function. A pre-transplant donor risk classification system based on CDKN2A and ECD criteria is shown to be feasible and commendable for implementation in the near future.

## Introduction

Kidney transplantation is the optimum treatment for renal failure but is restricted by donor shortage. A large proportion of End Stage Renal Failure (ESRF) patients must therefore receive alternative replacement therapies in the form of peritoneal dialysis, or haemodialysis. Such treatment results in increasing morbidity particularly affecting the cardiovascular system, a severely reduced lifespan and poorer quality of life. “Extended Criteria Donor” (ECD) kidneys are increasingly used to meet this shortfall in kidney supply.

In accordance with the Organ Procurement and Transplantation Network (OPTN) and United Network for Organ Sharing (UNOS), an Expanded Criteria Donor (ECD) is one which is: [1].

- 60 years or over
- 50–59 years with at least 2 of the following three medical criteria
  - Cerebro-Vascular Accident as the cause of death
  - History of hypertension
  - Pre retrieval creatinine more than 133 µmol/L

Although ECD organs incur elevated risks of Delayed Graft Function (DGF) and ultimately have unfavorable long-term outcomes compared with younger donor kidneys, average results remain far superior to alternative treatment modalities, such as haemodialysis. Some grafts, however, perform poorly – or never function adequately – and thus display Primary Non Function (PNF). The reasons for this phenomenon are unclear, but seem likely to relate to the inability of older kidneys to tolerate and recover from the multiple injurious processes associated with transplantation. In essence, such organs will have more ‘miles on the clock’ and thus not function as well, or last as long. The presence of substantial cellular senescence will make them more susceptible to the effects of transplant-related stresses. [2], [3] In general, however, poor function is difficult to predict as many older organs perform adequately despite advanced chronological age. [4], [5] Dependent upon the numbers of senescent cells present in an organ, tissue integrity may be impaired and the capacity to withstand stress reduced. Furthermore, senescence-associated upregulation of pro-inflammatory cytokine gene expression may lead to chronic persistent inflammation. We have therefore hypothesised that the biological age of the organ, rather than just its chronological age, may have a major impact on allograft function and that this may be directly relevant to discriminating between ECD organs.

This would imply that the expression of genes involved in cellular processes regulating biological ageing, should provide suitable reporters for investigating such a hypothesis. Indeed, robust and reproducible studies have shown that gene expression of senescence markers in a donor organ (organ bioage), can predict renal function in vivo, irrespective of classical parameters currently in use, such as donor chronological age and sub optimal pre-retrieval serum creatinine [6], [7].

To date, of those putative biomarkers of ageing (BoA) that have been tested, very few meet the Baker and Sprott criteria required for validation. [8] This dictates that a valid BoA must demonstrate variation of sufficient magnitude in short-term longitudinal, or in cross-sectional studies, to be of predictive value within a population or cohort with regard to physiological capacity at a later chronological age, in the absence of disease. [9] Failures include Senescence Associated β Galactosidase (SA-β-GAL), advanced glycation end products and lipofuscin, which were originally supported by substantial in vitro evidence. [10] In vivo, only two BoA have been validated with respect to renal function: Cyclin Dependant Kinase 2A (CDKN2A) and telomere length [6], [7].

Telomeres are nucleo-protein complexes at the ends of chromosomes with a DNA component comprising variable lengths of a TTAGGG simple repeat. Their primary role includes maintaining stability and protecting the integrity of chromosomes. [11] In somatic cells telomeric DNA shortens in length as a consequence of the end replication problem. [12] The rate of telomere shortening is directly influenced by oxidative stress. [13] This provides a rationale for using telomere length as a BoA at the cellular level and potentially explains the impact of environmental and lifestyle factors on inter-individual differences in the rate of ageing, [14] though with the caveat that it acts as a proxy for the effects of stress and not causal for it [15].

CDKN2A expression is a key age-related component of senescence in human renal allografts and renal disease. [16], [17] CDKN2A expression is elevated as a function of increasing cellular stress and organismal ageing. As such, this typically accompanies the telomere shortening observed during normal human ageing. CDKN2A acts as a tumour suppressor, is a component of STASIS (stress and stimulation induced senescence) [18] and is functionally involved in maintaining cells in a state of growth arrest. It has previously been demonstrated to be a significant pre-transplant predictor of post transplant renal allograft function [6], [7], [19].

In this study, we have sought to directly compare the expression of CDKN2A and telomere length in pre-implantation, time zero biopsies and correlate this with renal function up to 1 year post-operatively. We have sought to determine associations with donor chronological age and other important clinical variables in both univariate and multivariate regression analysis. Included in this analysis was renal function, assessed using the 4 variable “Modification of Diet in Renal Disease Study Group” formula - MDRD 4 eGFR (ml/min/1.73 m^2^), referred to as eGFR in the subsequent text. These analyses were designed to provide a basic indication of the importance of each respective BoA and to assess their capacity pre-transplant to predict post-transplant function and any associated adverse clinical characteristics, when used either singly, or in combination. Any indication of suitability in this respect could then be exploited, to provide a simple pre-transplant scoring or classification system by assessing BoA expression in the allograft as it is being cross-matched.

## Results

### Association between Biological Age and Chronological Age

**Figure 1:**
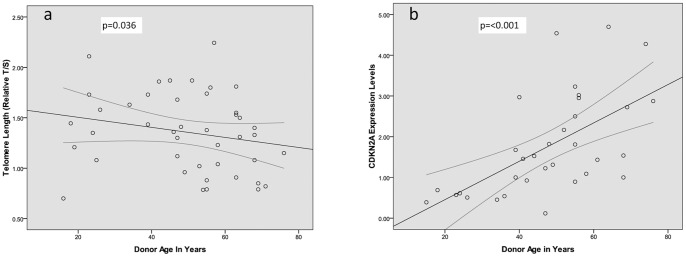
Scatter plots showing the correlation between biomarkers of ageing and donor chronological age. a. Negative correlation between Donor Chronological Age and Telomere Length. n = 43, CC: −0.242, p = 0.036. b. Positive correlation between Donor Chronological Age and CDKN2A. n = 33, CC: 0.597, p<0.001.

Prior to analysing the predictive power of biomarkers of ageing on renal function, data were validated by determining the association between telomere length and CDKN2A. A Pearson correlation between the two revealed no statistical significance (p = 0.87, n = 15). Telomere length and CDKN2A were then separately correlated with donor chronological age. Telomere length was shown to inversely correlate with chronological age (p = 0.036, CC = −0.242, Figure 1a), while CDKN2A levels positively associated with increasing chronological age (p<0.001, CC = 0.597, Figure 1b). These findings indicate that CDKN2A is more robustly associated with the chronological ageing process in kidney tissue when compared to telomere length. There was no difference in demographic and clinical data between both CDKN2A and telomere groups (Table 1).

**Table 1:** Demographic and important clinical parameters were compared between the separate CDKN2A group and the telomere group.

	CDKN2A n = 33 Mean (SD)	Telomere n = 43 Mean (SD)	p value
**Donor Gender** ** **(male/female)**	15/18	20/23	0.589
**Donor Age** **	48.0 (15.7)	51.8 (15.51)	0.189
**DCD/DBD organ** *	4/29	9/34	0.677
**Mismatch at all A,B,DR Loci** * **(yes/no)**	8/25	10/33	0.607
**Recipient Age** **	50.6 (12.7)	49.7 (12.6)	0.344
**Cold Ischaemic Time** **	15.5 (3.9)	13.9 (4.0)	0.267

There were no significant differences between the two groups which would account for the different correlations with renal function (eGFR).

** Unpaired t-Test.

* Fisher’s exact test.

### BoA and Correlation with Renal Function Post-Transplant

**Figure 2:**
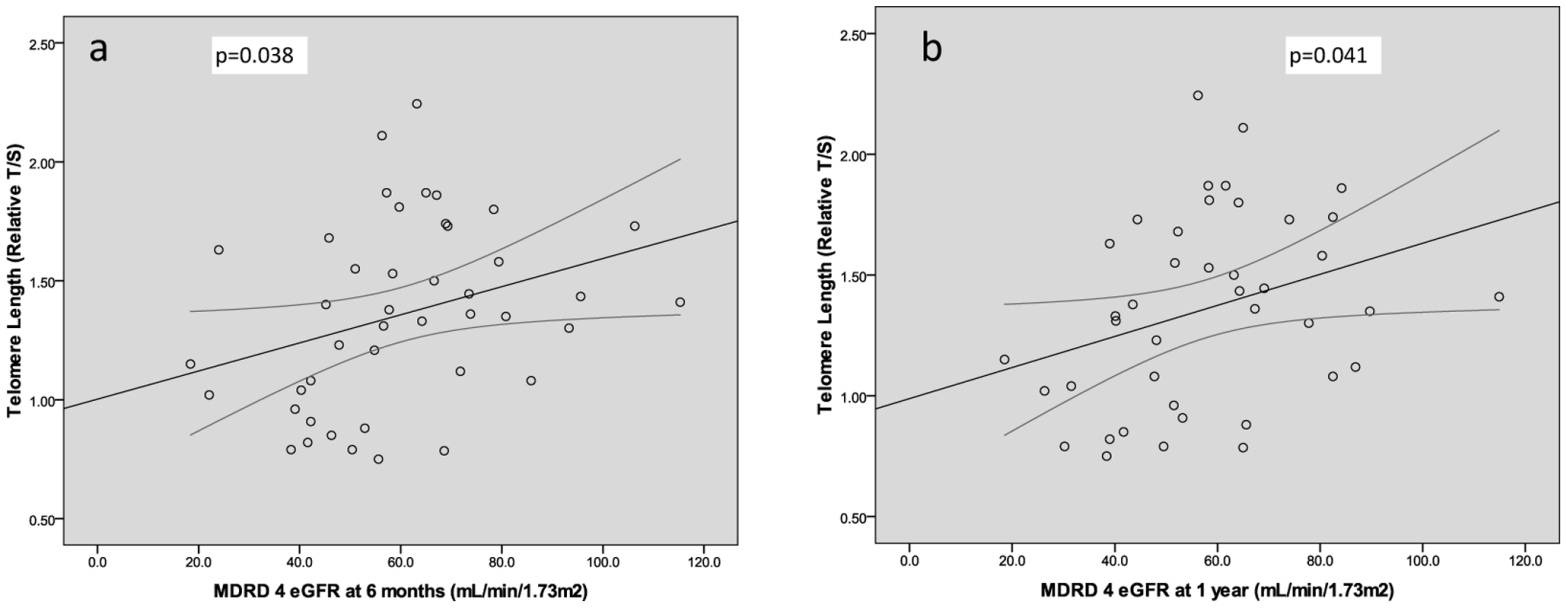
Scatterplots showing the primary significant relationship between telomere length and renal function, as measured by MDRD 4 eGFR at a) 6 months and b) 1 year. a. Telomere Length vs MDRD 4 eGFR at 6 months: n = 43, CC: 0.317, p = 0.038, b. Telomere Length vs MDRD 4 eGFR at 1 year: n = 41, CC: 0.320, p = 0.041.

**Figure 3:**
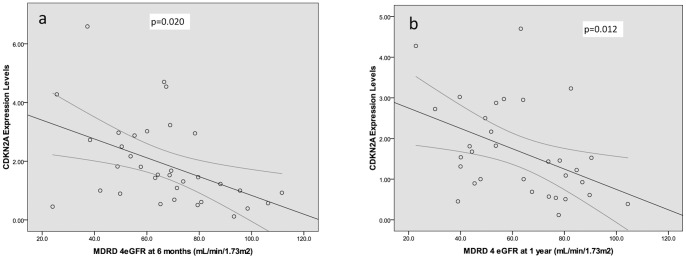
Scatterplots showing the primary significant relationship between CDKN2A and renal function, as measured by MDRD 4 eGFR at a) 6 months and b) 1 year. a.CDKN2A vs MDRD 4 eGFR at 6 months. n = 33, CC: −0.403, p = 0.020. b.CDKN2A vs MDRD 4 eGFR at 1 year. n = 32, CC: −0.439, p = 0.012.

Pearson correlation showed a significant association between shortening telomere length and deteriorating eGFR at 6 months and at 1 year post-transplant (p = 0.038 & p = 0.041, Figure 2). However, increasing levels of CDKN2A expression were associated with decreasing eGFR levels at 6 months and 1 year post-transplant (p = 0.020 & p = 0.012, Figure 3).

### Univariate Linear Regression

Univariate linear regression was used in order to assess the individual predictive power of multiple variables on eGFR. Each variable was used to predict function at both 6 months (Table 2) and 1 year (Table 3) post transplantation. The results indicated that CDKN2A, ECD, and donor chronological age were the strongest univariate predictors for eGFR. Telomere length in contrast displayed a poorer predictive ability in general. As expected, other clinical variables that are included in ECD criteria (donor age, donor hypertension, death by CVA but not high serum creatinine) significantly predicted eGFR at both timelines. Interestingly, there was a small but significant association for recipients who suffered any form of glomerulonephritis (GN) resulting in end stage renal failure. Recipients with ESRF second to GN displayed poorer renal function at both timelines (MWU - 6 months p = 0.05, 1 year p = 0.04). Important univariate associations displayed in Table 2 and 3 include:

**Table 2:** Univariate linear regression analysis showing the predictive power of CDKN2A, telomere length and other relevant clinical variables on renal function at 6 months.

Variable	MDRD 4 eGFR at 6 months		
	n	Adjusted R^2^	p-value
CDKN2A expression	33	0.135	0.020
Telomere Length	43	0.079	0.038
Donor Chronological Age	120	0.143	<0.001
GN in recipient	112	0.029	0.040
ECD Kidney	118	0.121	<0.001
Donor Hypertension	107	0.051	0.011
CVA in Donor	111	0.057	0.007
Donor pre-retrieval Creatinine >133 µMol/L	110	−0.008	ns
Mismatch at A, B and DR Loci	114	−0.009	ns
Previous Transplant	120	0.000	ns
Cold Ischaemic Time	114	0.019	ns
Donor Sex	120	−0.001	ns
DCD/DBD	63	−0.003	ns

Note the superior predictive strength of CDKN2A when compared to telomere length. (GN: Glomerulonephritis, DCD: Donation after Cardiac Death, DBD: Donation after Brain Death, CVA: Cerebro Vascular Accident, ECD: Extended Criteria Donor).

**Table 3:** Univariate linear regression analysis showing the predictive power of CDKN2A, telomere length and other relevant clinical variables on renal function at 1 year.

Variable	MDRD 4 eGFR at 1 year		
	n	Adjusted R^2^	p-value
CDKN2A expression	32	0.166	0.012
Telomere Length	41	0.079	0.041
Donor Chronological Age	104	0.214	<0.001
GN in recipient	105	0.028	0.048
ECD Kidney	103	0.174	<0.001
Donor Hypertension	100	0.069	0.005
CVA in Donor	95	0.075	0.004
Donor pre-retrieval Creatinine >133 µMol/L	95	−0.011	ns
Mismatch at A, B and DR Loci	98	−0.010	ns
Previous Transplant	104	0.000	ns
Cold Ischaemic Time	98	0.014	ns
Donor Sex	105	−0.009	ns
DCD/DBD	49	0.001	ns

Note again the superiority of CDKN2A over telomere length in particular. (GN: Glomerulonephritis, DCD: Donation after Cardiac Death.

DBD: Donation after Brain Death, CVA: Cerebro Vascular Accident, ECD: Extended Criteria Donor).

#### 6 months

Donor chronological age predicted 14.3% of the variability in eGFR whilst ECD kidney category predicted 12.1%. CDKN2A predicted 13.5% of the eGFR, whilst telomere length predicted 7.9%.

#### 1 year

Donor chronological age predicted 21.4% of the eGFR whilst ECD kidney category predicted 17.4%. CDKN2A predicted 16.6% of the eGFR, whilst telomere length remained at 7.9%.

### Multivariate Linear Regression Analysis

A multivariate regression model encompassing the three principle pre-transplant variables was formulated using eGFR as the dependant variable. The covariates were based on CDKN2A and the stronger clinical univariate predictors: ECD and presence or absence of glomerulonephritis in the recipient. Since donor hypertension, donor chronological age and death by CVA are already included under ECD criteria, they were not included as separate covariates in the model. The addition of telomere length to any model severely weakened it’s associations with renal function resulting in a statistically insignificant outcome. A total of two models were formulated at 6 months and 1 year timelines with a p-value of <0.017 taken to be statistically significant using Bonferroni’s correction. At 6 months, the model approached statistical significance (p = 0.021) as outlined in Table 4. Statistical significance was reached at 1 year where the model predicted 27.1% of the eGFR (Adjusted R^2^ 0.271, n = 31, p = 0.008 ANOVA) with respective individual contributions outlined in Table 5.

**Table 4:** Multivariate model outcome for eGFR at 6 months.

Independent Variable	Standardised Coefficients (Beta)	p-value
**CDKN2A**	−0.397	0.034
**ECD Kidney**	−0.211	0.233
**Recipient Glomerulonephritis**	−0.293	0.088

The model approaches statistical significance using the strict Bonferroni correction (p = 0.021).

**Table 5:** Multivariate model outcome for eGFR at 1 year.

Independent Variable	Standardised Coefficients (Beta)	p-value
**CDKN2A**	−0.428	0.019
**ECD Kidney**	−0.236	0.166
**Recipient Glomerulonephritis**	−0.311	0.061

The model explains 27.1% of the eGFR p = 0.008.

### CDKN2A, Delayed Graft Function and Rejection

Increased expression of CDKN2A in pre-implantation biopsies was significantly associated with DGF (MWU, p = 0.032). Median CDKN2A expression levels in patients with DGF were compared with those grafts that showed primary function (DGF CDKN2A mean expression = 2.61 (SD 0.56, n = 6) vs primary function CDKN2A mean expression = 1.61 (SD 1.30, n = 27)). DGF in itself was significantly correlated with graft rejection episodes (Fisher’s exact test, n = 113, p = 0.001). This data suggests that high levels of CDKN2A are linked to increased allograft immunogenicity resulting in increased rejection episodes in the long term and may also play a role in the aetiology of DGF through a similar mechanism.

A total of 112 patients with rejection data were analysed. Biopsy proven evidence of acute rejection was present in 25.0% (n = 28) of the total. All such grafts were viable at 6 months with a median eGFR of 39.05 ml/min/1.73 m^2^ (SD 17.16) however, there was 1 graft failure at 1 year, with a median eGFR for all other grafts (n = 27) of 39.70 ml/min/1.73 m^2^ (SD18.09). There was no direct statistical relationship between CDKN2A and rejection itself (p = 0.741).

## Discussion

We have demonstrated that the pre-transplant expression of two independent BoAs correlates with renal function post-transplant. Greater biological age, as determined by shorter telomere length, or higher relative CDKN2A expression, correlated with poorer post-transplant function [19]. This is in keeping with observations in the field. Classically, organs from older donors show poorer function post-transplant and have a decreased lifespan. Although this holds true in most cases, there are times when such organs perform very well and last beyond their life expectancy. Our results indicate that such variation in organ function could be attributed to the difference in biological age. Our data indicate that pre-transplant CDKN2A expression is the strongest biomarker of renal function up to 1 year post-operatively. When used in the context of Baker and Sprott’s criterion, CDKN2A appears to be significantly more robust as a BoA than telomere length. The latter may be viewed as an effective but imprecise BoA. Distinguishing between age-related telomere attrition and disease-related attrition is difficult [8]. Using both together as a composite measure, alongside chronological age, should be of further benefit in this context. Clinical translation of this should be straightforward, as our methodology is readily adaptable to implementation when the organ is undergoing cross-match.

In comparison to previous studies, we used the estimated Glomerular Filtration Rate (eGFR) as a marker for renal function as it is traditionally considered the best overall index of function in health and disease. [20] The National Kidney Foundation now recommends the MDRD 4 to estimate the GFR and better detect early onset kidney disease. Although the eGFR is considered to be the best overall index of renal function, it is relatively insensitive at detecting early renal disease and does not correlate well with tubular dysfunction [21], [22].

We have previously shown that CDKN2A is stronger than donor chronological age (DCA) at predicting post transplant function when serum creatinine is used as the marker for renal function. [7] However, when eGFR is used to measure renal function, DCA seemed to have a better predictive power than CDKN2A (Tables 2 and 3). Further univariate regression analysis revealed that the predictive power of CDKN2A on eGFR was almost equal to that of ECD kidney criteria (Tables 2 and 3). In multivariate analysis, the only statistically significant contribution to both models is CDKN2A, indicating it’s predictive superiority in this limited cohort.

Despite increasing efforts by the transplant community to increase the availability of donor organs, there remains a significant shortfall with several thousand patients dying on the waiting list each year. The introduction of ECD kidneys has improved the quantitative discrepancy of such organs but we are still a distance from achieving satisfactory targets. Novel techniques of organ discrimination are therefore of huge importance in this respect. With the standard incorporation of biomarkers in assessing organ quality pre-operatively, it would seem logical that transplantation would be safer and an increase in the number of kidney transplants would subsequently ensue. CDKN2A is also related to DGF which in itself is associated with poorer graft performance and decreased long term survival. [23], [24] The reason for this remains to be determined, but may relate to biologically older organs being less tolerant to physical stress and requiring more time to recover from peri-transplant ischaemia reperfusion injury.

Why CDKN2A expression levels, in this study, have been observed to be a stronger biomarker of ageing than telomere length remains to be proven. Both fulfil the Baker and Sprott criterion, but the weakness of telomere length in predicting functional capacity in a solid organ is apparent. A contributory factor may be the extent of inter individual variation in telomere length at a given chronological age. [6], [8], [14] Our data are consistent with those of Koppelstaetter et al [6], who previously demonstrated that telomere length was inferior to CDKN2A in determining variability on post-transplant serum creatinine levels in renal allografts. Inter-individual variation in CDKN2A expression at a given chronological age has not been fully determined, though increased expression of CDKN2A at the cellular level, remains a robust marker of a senescent state and its elevated expression is coincident with a reduction in cellular proliferation. [25] In essence, its expression may be viewed as an ‘off switch’ for the cell and hence the degree of inter-individual variation observed with telomere length, is not expected to be as great. Our observations have direct relevance for any future strategies employing biomarkers of ageing either clinically, or epidemiologically. Telomere length is currently used widely in this context. We are now evaluating CDKN2A similarly, in large epidemiological studies, to evaluate its robustness with greater analytical power.

Based on current findings relating to the predictive power of CDKN2A on eGFR, it would follow that a scoring system incorporating biological markers would provide additional information for patients and clinicians during the organ selection process. Reference is made to larger studies such as the one in use by the OPTN in the US for deceased donor kidneys based on ten pre-transplant covariates, the Kidney Donor Risk Index. [26] Undoubtedly, this novel scoring system adds a vital tool to the allograft allocation process. Importantly however, it does not include reference to biological age which may be viewed as an essential parameter of modernised scoring systems. In addition, the study itself showed similar results with age matching alone allowing for the possibility of a simpler scoring technique with equal efficacy. We therefore propose a 4 tier categorical scoring system based on biological age of the graft and ECD. Allografts are classified Category I to Category IV based on a straight forward assessment outlined below, with Category I allografts predicting better performance than Category 4 (Table 6).

**Table 6:** Suggested Donor Kidney Classification system incorporating CDKN2A as the biomarker of ageing and ECD kidney criteria.

**Category I**	SCD Kidney and CDKN2A expression levels <1.8
**Category II**	SCD Kidney and CDKN2A expression levels >1.8
**Category III**	ECD Kidney and CDKN2A expression levels <1.8
**Category IV**	ECD Kidney and CDKN2A expression levels >1.8

(SCD – Standard Criteria Donors, ECD – Extended Criteria Donors). Predicted kidney function and incidence of graft failure increases with higher category placement.

The mean value for CDKN2A gene expression (1.8) was used as the cut-off value in the scoring system. Moreover, it can be seen from the scatter plots of CKDN2A vs eGFR at 1 year that renal function deteriorates significantly at CDKN2A expression levels above 1.8. ECD kidneys occupy both category III and category IV in this pre-transplant scoring tool meaning that ECD status carries a poorer prognosis than CDKN2A itself. The allocation of CDKN2A to a higher tier in this scoring system would require further studies to strengthen the correlations observed above. Since DCA forms part of ECD criteria, it was not used as a single determinant of transplant function in multivariate analysis or the categorical scoring system.

A further benefit from our data, is that strategies to mitigate the rate of biological ageing applied to living donors would be expected to have impact on post-transplant outcomes. Reduction of psychological and psychosocial stress and improved lifestyle via changes to diet and exercising might readily be considered. [14], [27], [28] Biomarkers, specifically CDKN2A, may well expand the field of octogenarian donation for example, by discriminating organs with “less miles on the clock”. Larger multicentre studies are needed to strengthen the hypothesis and the proposed scoring system suggested in this report. It is envisaged that the biomarker CDKN2A will be integrated into a similar, robust and validated pre-transplant scoring system for all kidneys and other transplanted organs in the near future.

## Methods

### Ethics

This is an ongoing prospective study which has been approved by the Regional Ethics Committee of the North Glasgow NHS Trust. Donors from the national pool donated their organs for transplantation. The recipient of the organ provided pre-operative written informed consent for tissue analysis and scientific research. Samples were anonymised and subsequently analyzed.

### Study Population

The global study population is representative of the national UK deceased donor pool. A total of 120 transplant patients were included which were performed in the Western Infirmary, Glasgow between March 2008 and February 2011. All transplants were followed up for post-operative clinical data. Telomere length was calculated for 43 transplanted kidneys. A separate group (n = 33) yielded CDKN2A expression. There were 15 matched samples as a result of small biopsy specimens allowing RNA or DNA to be obtained separately and not together. Table 1 shows the demographic data for the CDKN2A and telomere groups. Patients, in whom genetic data was not available, were included in the global cohort for clinical analysis. The primary cause of end stage renal disease (ESRF) in the recipients was Adult Polycystic Kidney Disease (APKD) followed by chronic pyelonephritis/reflux disease, hypertensive nephropathy, IgA nephropathy and the glomerulonephritides. The immunosuppressive regimen consisted primarily of basiliximab at induction and day 4 with a maintenance regime consisting of tacrolimus, mycophenolate mofetil and prednisolone.

### Human Renal Biopsies and RNA/DNA Extraction

Renal biopsies were obtained on the surgical backbench via wedge resection or needle biopsy according to the surgeon’s preference. All biopsies were obtained from “donation after brain death” (DBD) and “donation after cardiac death” (DCD) donors. All samples were stored in ‘RNA later’ solution (Ambion, Austin, TX, USA) at –20°C until processing. RNA was extracted using Trizol reagent (Invitrogen, Paisley, UK) following manufacturer’s guidelines. The Maxwell® 16 DNA purification robot kits by Promega were used for for DNA isolation.

### Delayed Graft Function (DGF)

DGF was defined as failure of serum creatinine to fall by half within seven days of the transplant, or need for dialysis within seven days of the transplant, except dialysis performed for fluid overload or elevated serum potassium levels [29].

### MDRD 4 eGFR

The final value for the eGFR was calculated electronically by a biochemical and clinical database – SERPR. The four variables used in the equation include serum creatinine, age, race and gender [20].

### CDKN2A Expression Determination

Relative quantitative real-time PCR (qRT-PCR) was used to estimate mRNA levels corresponding to the candidate senescence associated gene (SAGs) - CDKN2A. Expression levels were measured against a reference HPRT housekeeping gene on an ABI Prism® 7700 Sequence Detection System. Sequences of human TaqMan™ Primer/Probe sets designed by Primer Express algorithm (Applied Biosystems, Austin, TX, USA).

### HPRT

Forward Primer (5/−CTTGCTCGAGATGTGATGAAG-3/),

Reverse Primer (5/−CAGCAGGTCAGCAAAGAATTTATAG-3/),

Probe (5/−FAM-ATCACATTGTAGCCCTCTGTGTGCTCAAGGTAMRA-3/)

### CDKN2A

Forward Primer (5/−CATAGATGCCGCGGAAGG-3/),

Reverse Primer (5/−CCCGAGGTTTCTCAGAGC-3/),

Probe (5/−FAM-CCTCAGACATCCCCGATTG-TAMRA-3/)

The comparative threshold cycle method (ΔΔCT) was employed as the method of choice to quantify relative gene expression. The quantification result was transformed to an exponential value, 2–ΔΔCt [30] where Ct is the threshold cycle, or the cycle when the product was first detected. Before undertaking this quantitative study we demonstrated that the efficiency of amplification of reference (HPRT) and test genes were approximately equal (data not shown).

### Telomere Length Determination

Telomere length determination was performed by qPCR using a Roche Light Cycler LC480. Telomere length analyses were performed in triplicate for each sample, using a single-copy gene amplicon primer set (acidic ribosomal phosphoprotein, 36B4) and a telomere-specific amplicon primer set. Quality control parameters employed for the amplifications comprised using a cut off 0.15 for the standard deviation (SD) of the threshold cycle (Ct) for sample replicates. At a SD above 0.15 the sample was reanalysed. The average SD across plates was <0.05.

Relative telomere length was estimated from Ct scores using the comparative Ct method after confirming that the telomere and control gene assays yielded similar amplification efficiencies. This method determines the ratio of telomere repeat copy number to single copy gene number (T/S) ratio in experimental samples relative to a control sample DNA. This normalised T/S ratio was used as the estimate of relative telomere length (Relative T/S).

### TELOMERE

Telo 1 Sequence (5′ to 3′)

CGG TTT GTT TGG GTT TGG GTT TGG GTT TGG GTT TGG GTT

Telo 2 Sequence (5′ to 3′)

GGC TTG CCT TAC CCT TAC CCT TAC CCT TAC CCT TAC CCT

36B4

36B4d Sequence (5′ to 3′)

CCC ATT CTA TCA TCA ACG GGT ACA A

36b4u Sequence (5′ to 3′)

CAG CAA GTG GGA AGG TGT AAT CC

### Statistics

Data analyses were performed using SPSS statistical package version 17. The adjusted R^2^ was used to indicate the extent to which the dependant variable (eGFR) is explained by the independent variable in question. The association was deemed to be statistically significant if the p value <0.05. Prior to multivariate regression, preliminary analysis was conducted to ensure no violation of the assumptions of normality, linearity and multicollinearity. Any missing values were removed by pairwise deletion. Bonferroni’s adjustment was used to calculate the exact p value for the multivariate models.
